# Supplementary material for: Tomography of the source zone of the great 2011 Tohoku earthquake
Source: Nat Commun. 2020 Mar 3;11:1163. doi: 10.1038/s41467-020-14745-8 (PMC7054414; doi:10.1038/s41467-020-14745-8)
Supplement: Supplementary file 1 — Supplementary Information [file 41467_2020_14745_MOESM1_ESM.pdf]

Supporting information for

Tomography of the source zone of the great 2011 Tohoku earthquake

By Hua et al.

Supplementary Figures

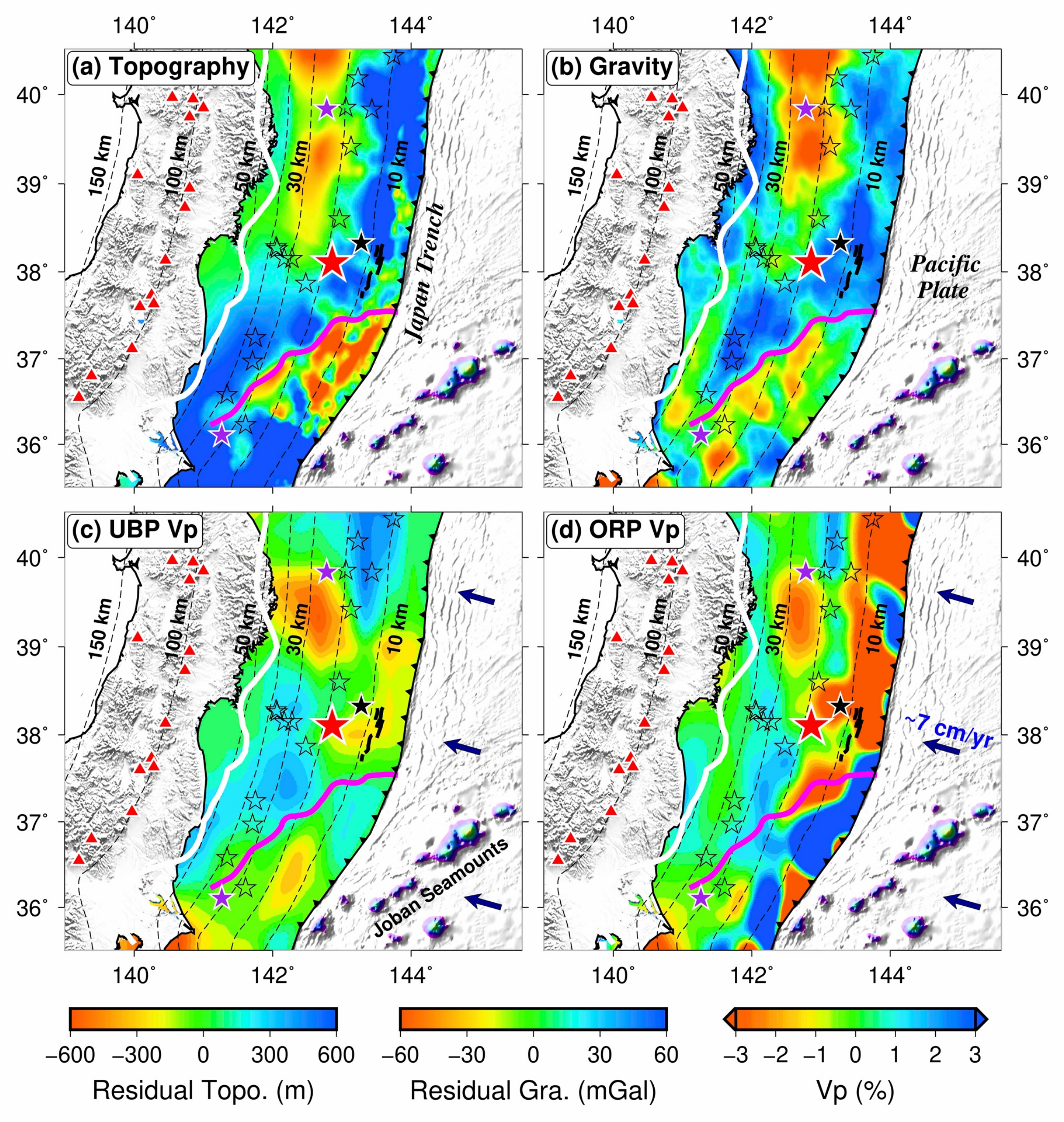

Supplementary Figure 1. Forearc anomalies in the Tohoku subduction zone. (**a**) Residual topography and (**b**) residual gravity derived from Bassett et al.[1]. The black dashed lines denote the upper boundary of the subducting Pacific slab (UBP). Red triangles denote active volcanoes. (**c**) Residual P-wave velocity (Vp) tomography along the UBP and (**d**) average 2-D Vp image of the overriding plate (ORP) obtained by this study. The magenta line marks the forearc segment boundary (FSB) proposed by Bassett et al.[1]. The white line denotes the estimated lower boundary of the Tohoku megathrust zone. The red star denotes the mainshock epicenter of the great 2011 Tohoku-oki earthquake (Mw 9.0), whereas the black open stars denote epicenters of other megathrust earthquakes (M ≥ 7.0) that occurred during 1917 to 2011. The black solid star denotes the Tohoku-oki earthquake foreshock (M 7.3) on March 9, 2011. The two purple stars denote large aftershocks (M ≥ 7.5) of the Tohoku-oki earthquake on March 11, 2011. Scales of the residual topography, residual gravity and residual Vp are shown at the bottom.

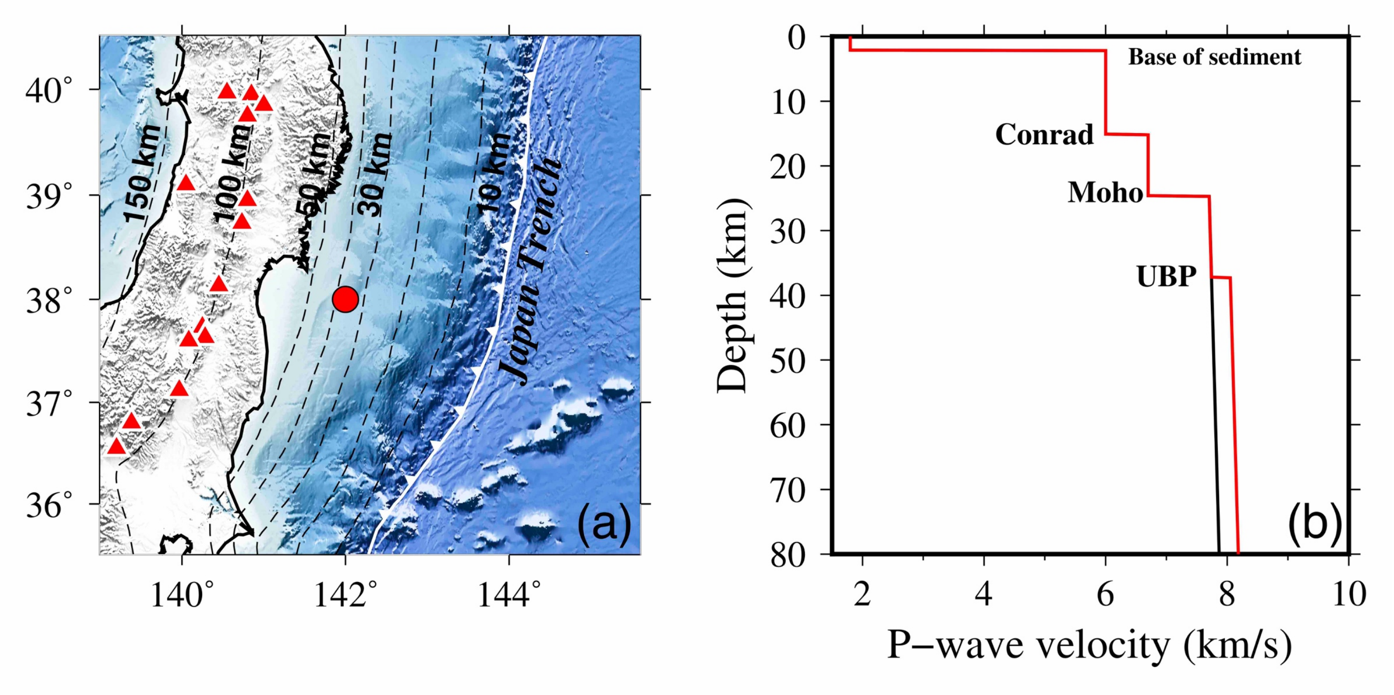

Supplementary Figure 2. 1-D P-wave velocity (Vp) model used in the present study. The red line in (b) denotes the 1-D Vp model at the red point shown in (a). The black line in (b) denotes the 1-D Jeffreys-Bullen Vp model.

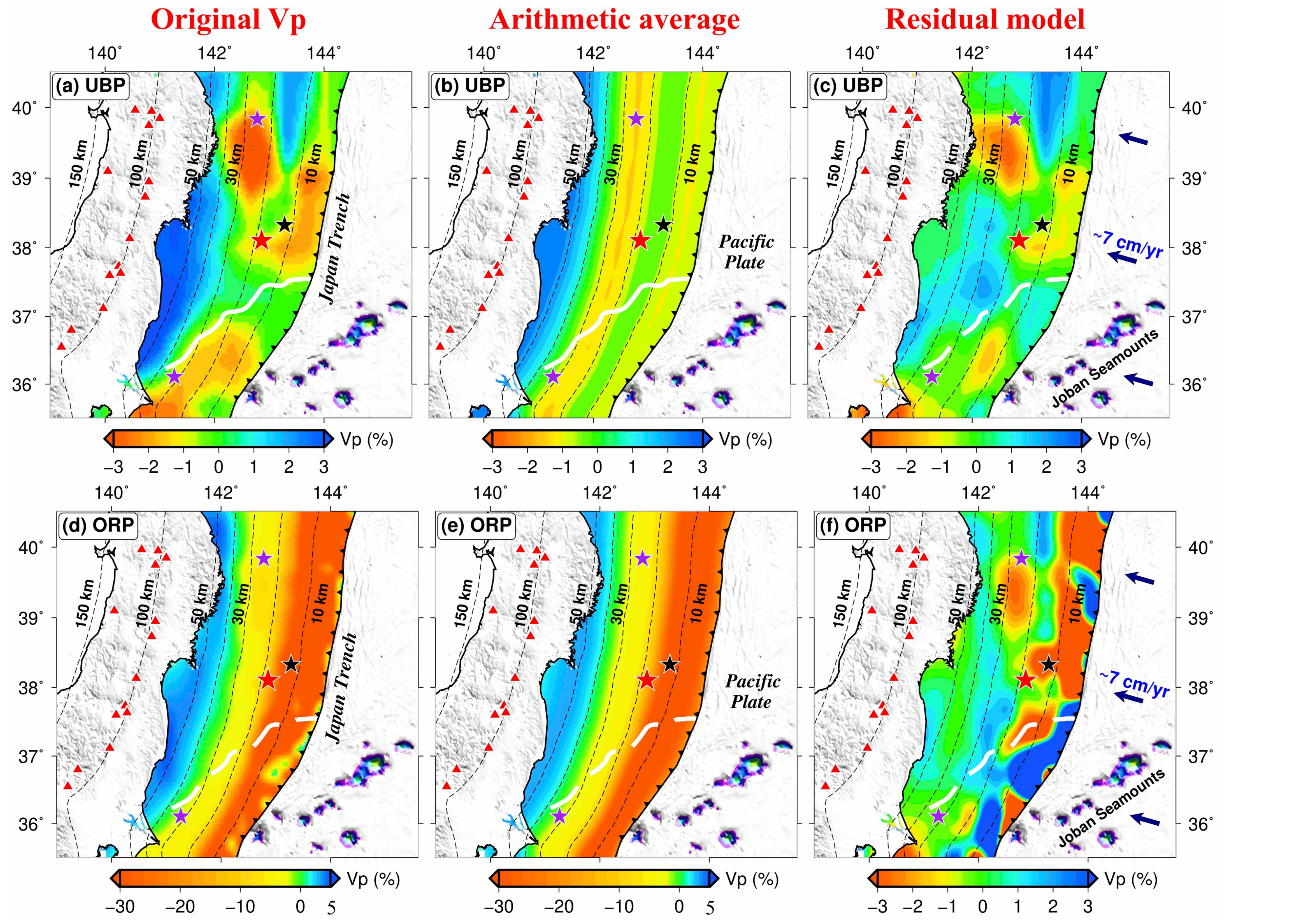
Original Vp
Arithmetic average
Residual model
(a) UBP
(b) UBP
(c) UBP
(d) ORP
(e) ORP
(f) ORP
140°
142°
144°
36°
37°
38°
39°
40°
150 km
100 km
50 km
30 km
10 km
Japan Trench
Pacific Plate
~7 cm/yr
Joban Seamounts
Vp (%)
−3 −2 −1 0 1 2 3
−30 −20 −10 0 5

Supplementary Figure 3. Calculation of residual Vp image. (**a**) Original Vp model along the UBP obtained by the tomographic inversion. Red and blue colors denote low and high Vp perturbations, respectively, relative to the 1-D Vp model as shown in **Fig. S2.** The black dashed lines denote depth contours of the UBP. The red star shows the epicenter of the 2011 Tohoku-oki earthquake (Mw 9.0) relocated by Zhao et al.[2]. The black star denotes the Tohoku-oki earthquake foreshock (M 7.3) on March 9, 2011. The two purple stars denote large aftershocks (M ≥ 7.5) of the Tohoku-oki earthquake on March 11, 2011. (**b**) The arithmetic average of the original Vp perturbations in (**a**) along each contour line of the UBP. The Vp arithmetic average of each contour line (**b**) is then subtracted from the original Vp model (**a**), resulting in the residual Vp model (**c**). Panels (**d** to **f**) are the same as panels (**a** to **c**) but for the Vp images of the overriding Okhotsk plate (ORP). The Vp perturbation scale is shown below each map.

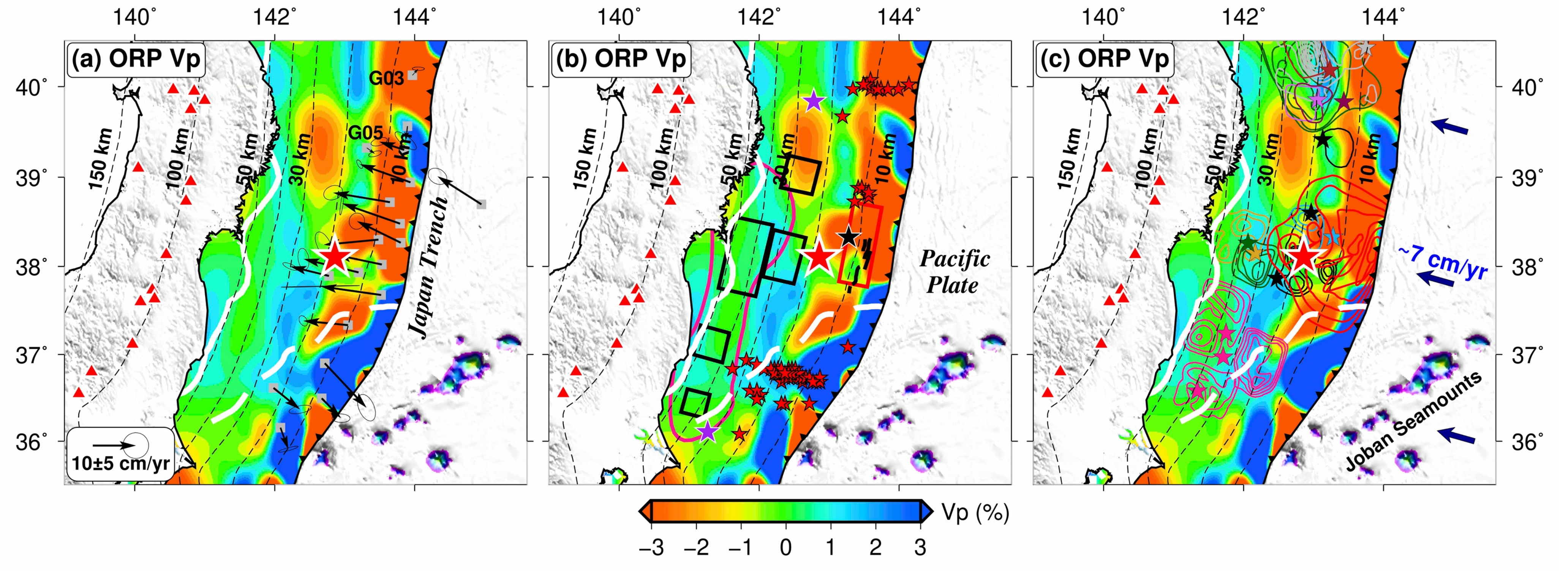

Supplementary Figure 4. Residual Vp tomography of the overriding plate and characteristics of the 2011 Tohoku-oki earthquake. The colors in (**a** to **c**) show residual Vp tomography of the overriding plate obtained by this study. Black arrows in (**a**) show the postseismic displacement rates with 1σ error ellipses estimated by Tomita et al.[3] whose observation period was from September 2012 to May 2016. (**b**) Black rectangles denote locations of coseismic strong ground motions[4]. The magenta contour line marks the site of coseismic high-frequency P-wave radiation with a relatively low seismic moment during the 2011 Tohoku-oki earthquake[5]. The red rectangle denotes a slow slip event (M 7.0) preceding the 2011 Tohoku-oki earthquake[6]. The small red stars denote epicenters of very low frequency earthquakes (VLFEs)[7]. The black short lines denote normal faults near the Japan trench. (**c**) The red contour lines near the trench denote coseismic slip distribution of the 2011 Tohoku-oki earthquake[8]. Other color contour lines and small stars show coseismic slip distributions and epicenters of large megathrust earthquakes[9] (M ≥ 7.0) that occurred during 1900 to 2011.

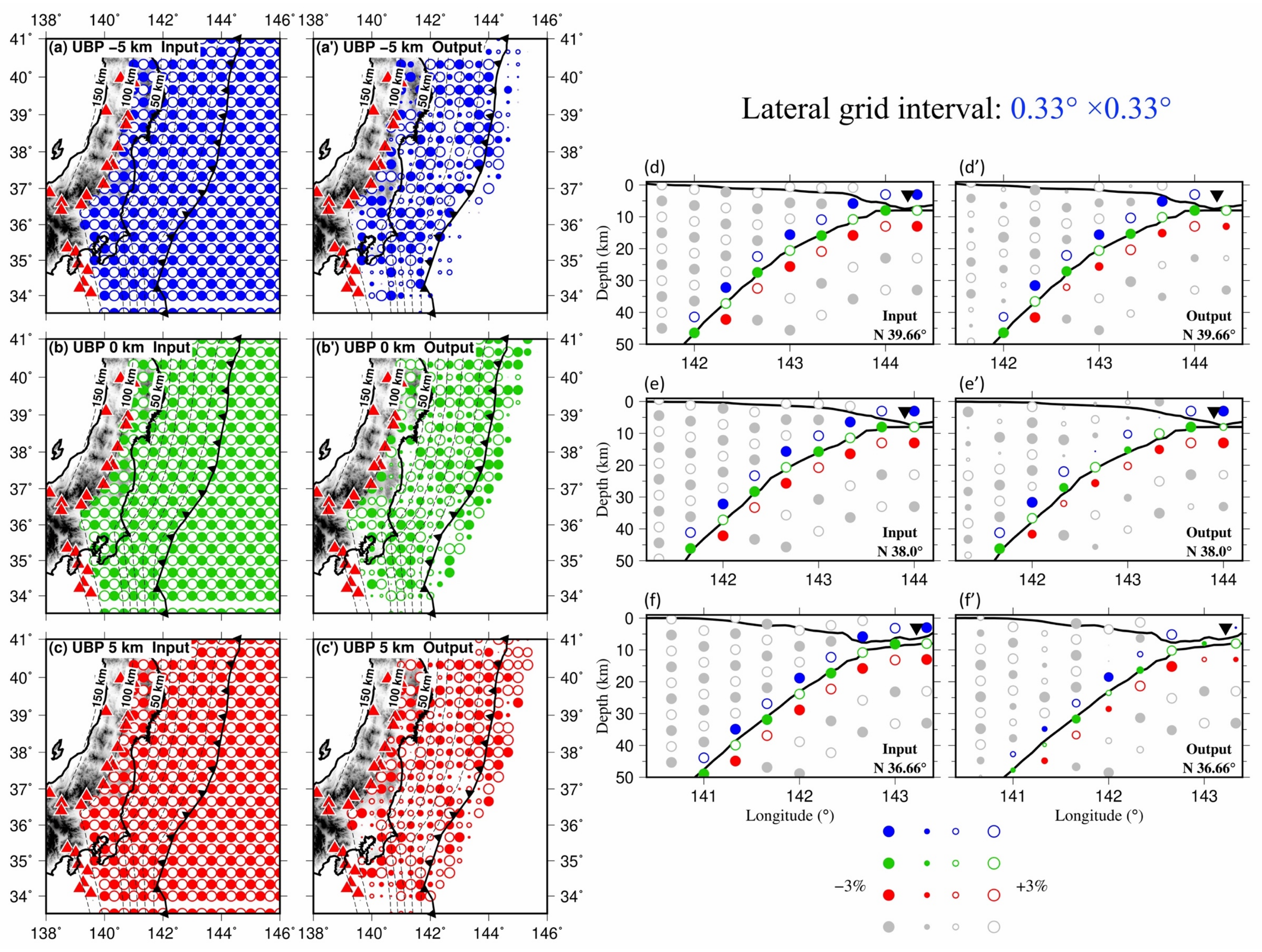

Supplementary Figure 5. Results of the first checkerboard resolution test (CRT) for Vp tomography. (**a** to **f**) show the input model, whereas (**a' to f'**) show the output results. The solid and open circles denote low and high Vp perturbations, respectively, whose scales (in %) are shown at the bottom. "UBP", "UBP -5 km" and "UBP +5km" denote three planes along the upper boundary of the subducting Pacific plate (UBP) and at depths of 5 km above and below the UBP, respectively. "39.66° N"," 38° N" and "36.66° N" denote vertical cross-sections along 39.66° north latitude, 38.0° north latitude and 36.66° north latitude, respectively. The black solid lines in each cross-section denote the surface topography and the UBP. The red triangles in the maps denote active volcanoes. The black reverse triangle in each cross-section denotes the location of the Japan Trench.

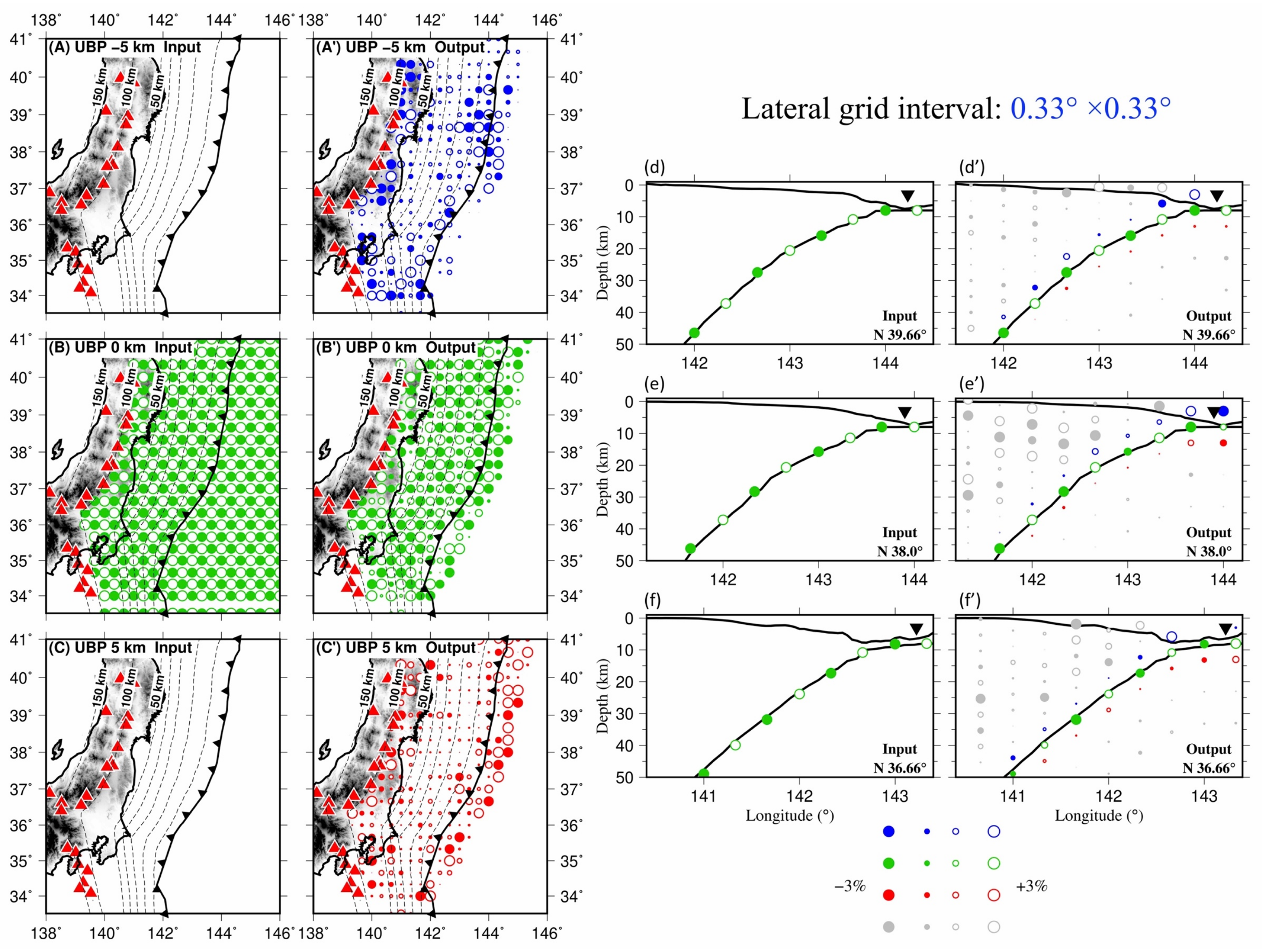

Supplementary Figure 6. Results of the second CRT for Vp tomography. This figure is the same as **Fig. S5** but the input model is different. In the input model, positive and negative Vp perturbations are only assigned to the grid nodes at the UBP.

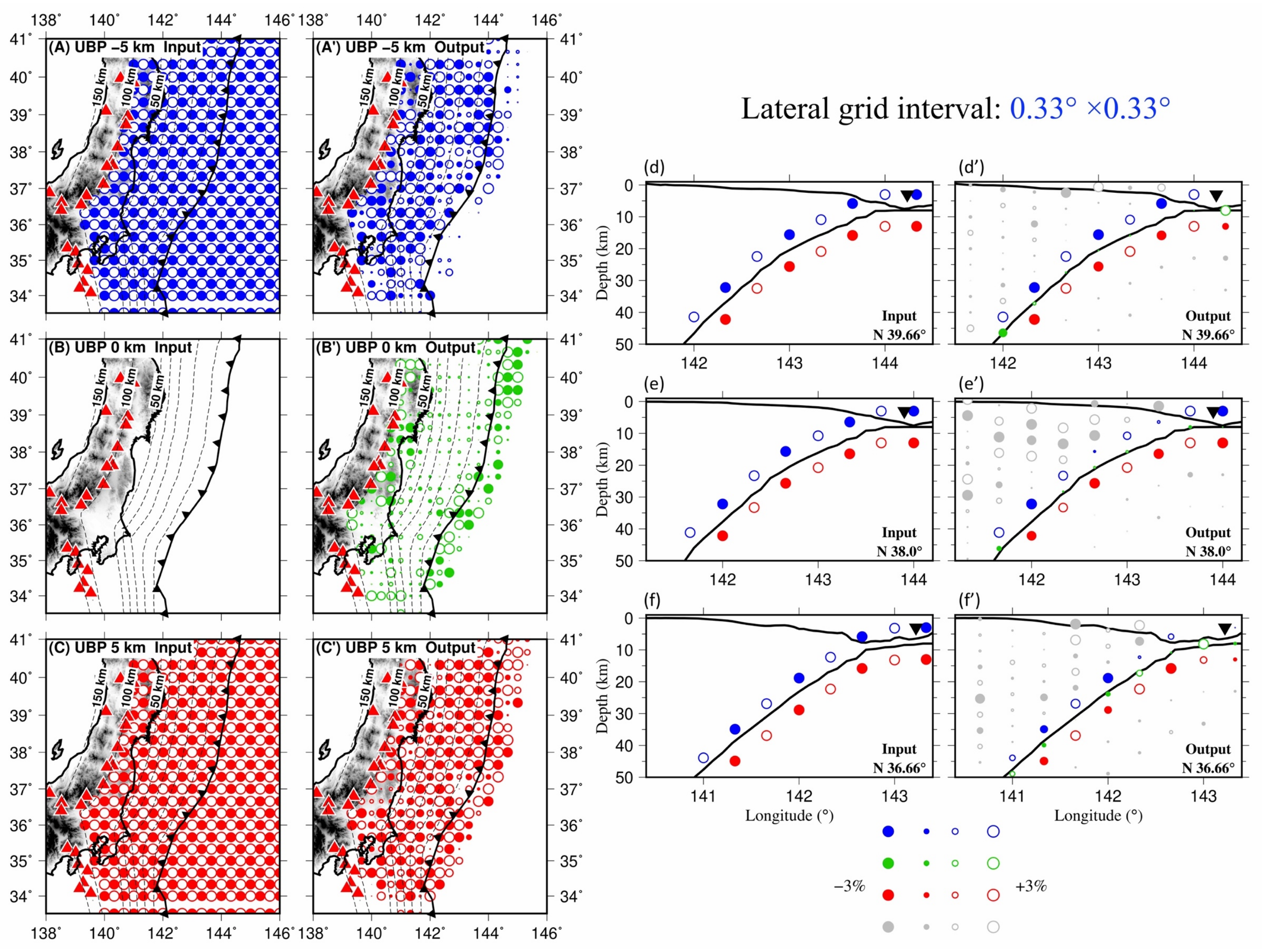

Supplementary Figure 7. Results of the third CRT for Vp tomography. This figure is the same as **Fig. S5** but the input model is different. In the input model, positive and negative Vp perturbations are only assigned to the grid nodes at depths of 5 km above and below the UBP.

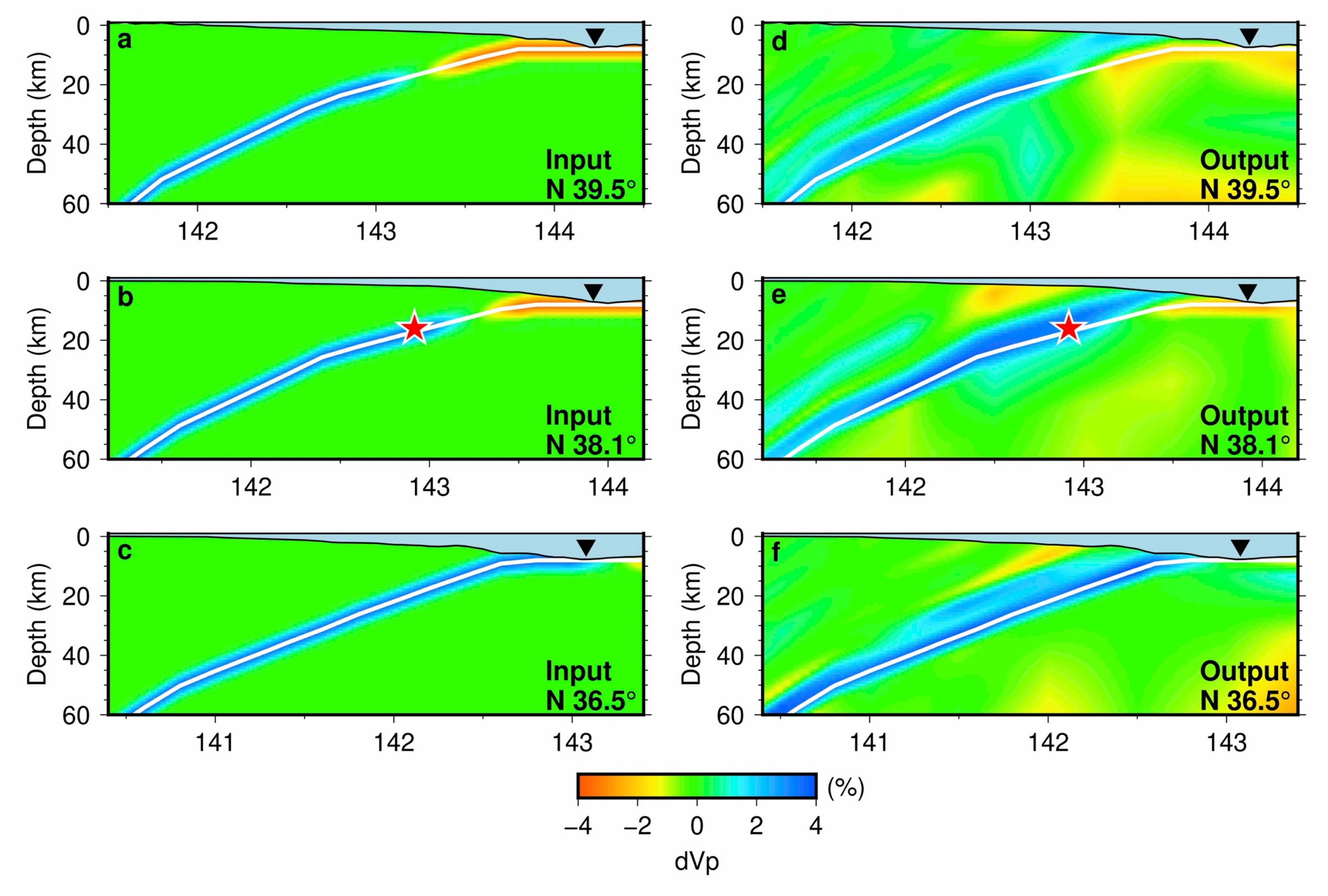

Supplementary Figure 8. Results of the first synthetic test for Vp tomography. Panels (**a** to **c**) show the input model, whereas panels (**d** to **f**) show the output results. Red and blue colors denote low and high Vp perturbations, respectively, whose scale is shown at the bottom. The red star denotes the hypocenter of the great 2011 Tohoku-oki earthquake (Mw 9.0).

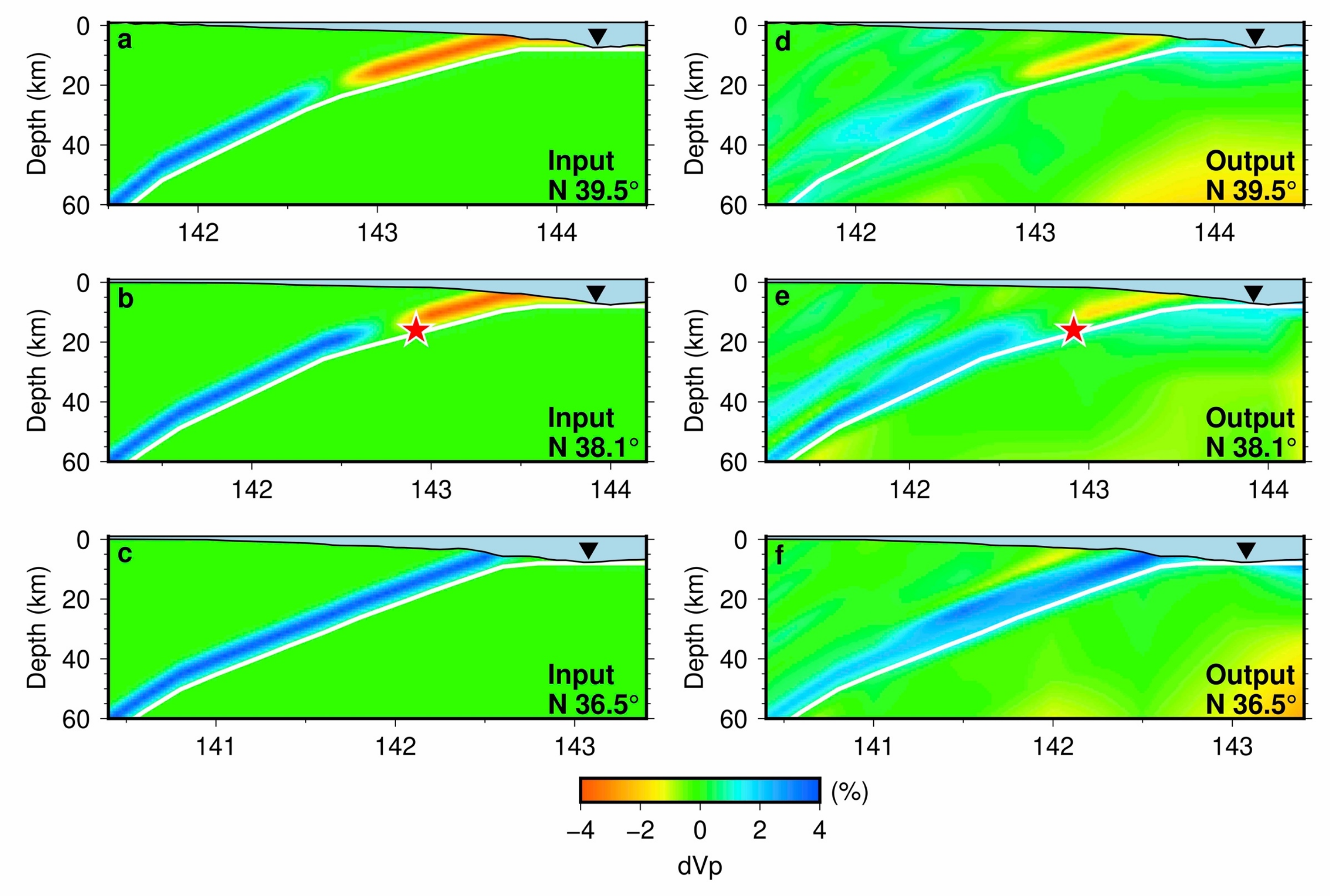

Supplementary Figure 9. Results of the second synthetic test for Vp tomography. Panels (**a** to **c**) show the input model, whereas panels (**d** to **f**) show the output results. Red and blue colors denote low and high Vp perturbations, respectively, whose scale is shown at the bottom. The red star denotes the hypocenter of the great 2011 Tohoku-oki earthquake (Mw 9.0).

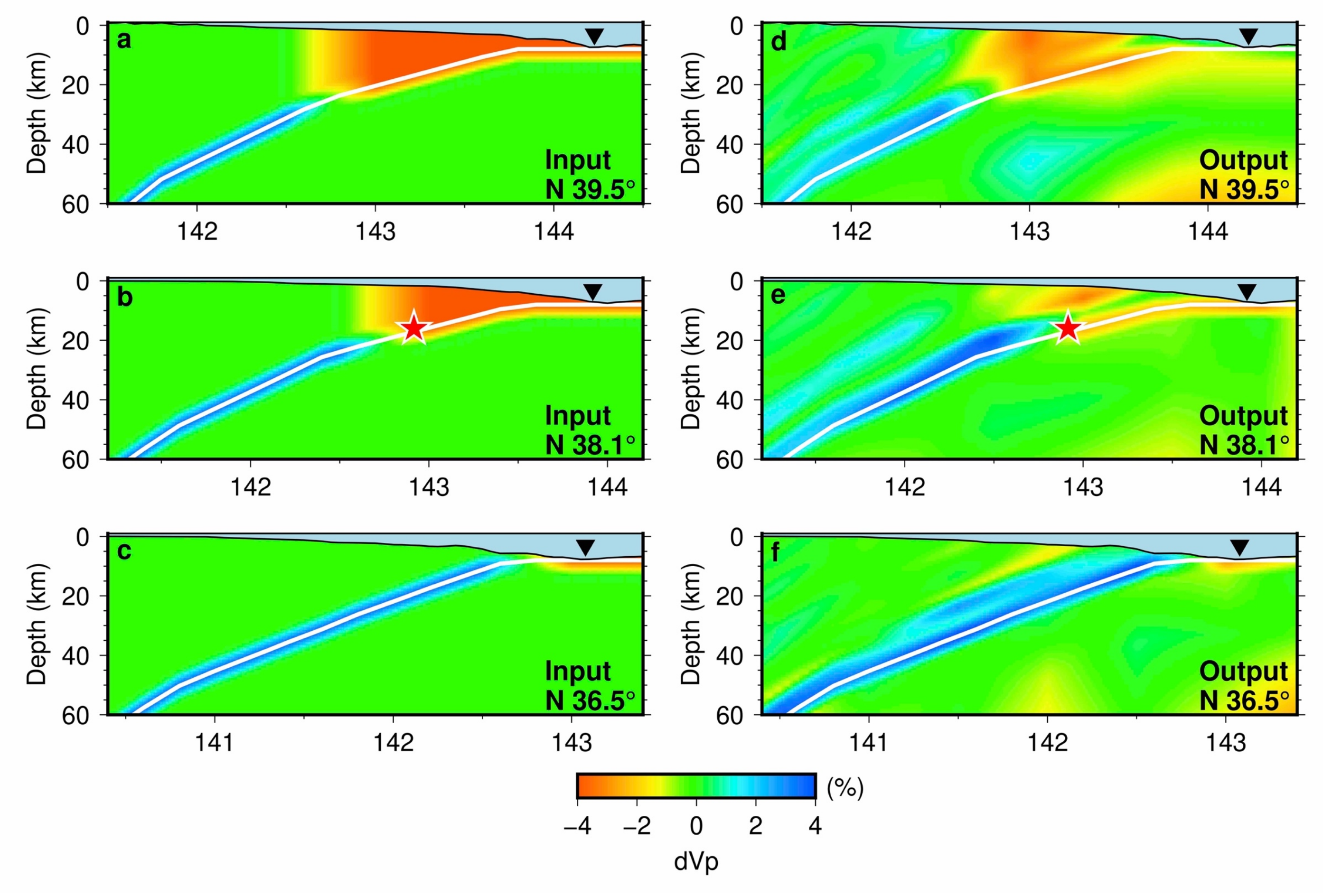

Supplementary Figure 10. Results of the third synthetic test for Vp tomography. Panels (**a** to **c**) show the input model, whereas panels (**d** to **f**) show the output results. Red and blue colors denote low and high Vp perturbations, respectively, whose scale is shown at the bottom. The red star denotes the hypocenter of the great 2011 Tohoku-oki earthquake (Mw 9.0).

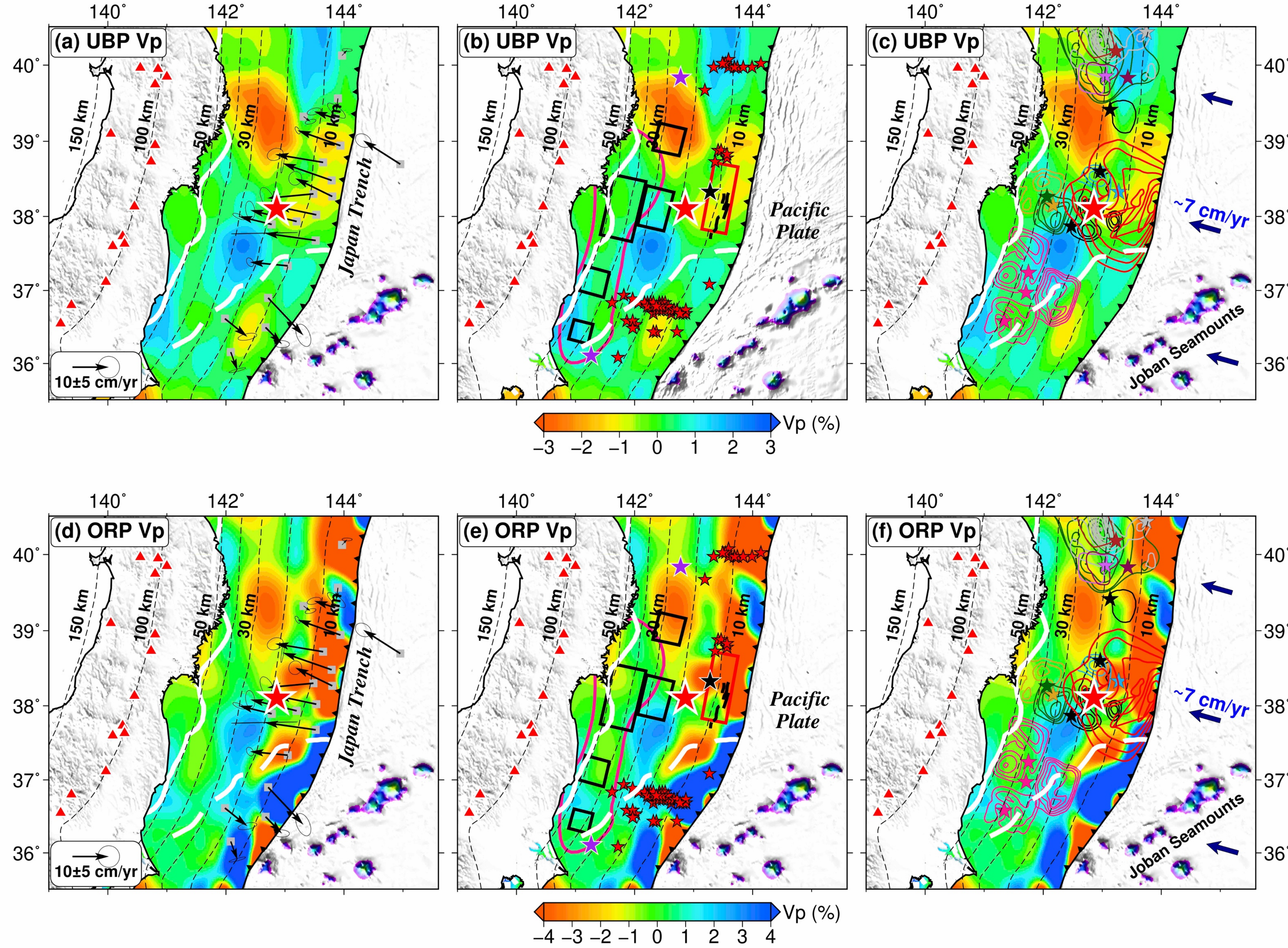
(a) UBP Vp
(b) UBP Vp
(c) UBP Vp
(d) ORP Vp
(e) ORP Vp
(f) ORP Vp
140°
142°
144°
36°
37°
38°
39°
40°
150 km
100 km
50 km
30 km
10 km
Japan Trench
Pacific Plate
~7 cm/yr
Joban Seamounts
10±5 cm/yr
Vp (%)
−3 −2 −1 0 1 2 3
−4 −3 −2 −1 0 1 2 3 4

Supplementary Figure 11. Residual Vp tomography determined by a joint inversion of our S-net data and the data set used by Liu and Zhao (2018). The colors in (**a** to **c**) show residual Vp tomography along the UBP. Black arrows in (**a**) show the postseismic displacement rates with 1σ error ellipses estimated by Tomita et al.[3] whose observation period was from September 2012 to May 2016. (**b**) Black rectangles denote locations of coseismic strong ground motions[4]. The magenta line marks the site of coseismic high-frequency P-wave radiation with a relatively low seismic moment during the 2011 Tohoku-oki earthquake[5]. The red rectangle denotes a slow slip event (M 7.0) preceding the 2011 Tohoku-oki earthquake[6]. The small red stars denote epicenters of very low frequency earthquakes (VLFEs)[7]. The black short lines denote normal faults near the Japan trench. (**c**) The red contour lines near the trench denote coseismic slip distribution of the 2011 Tohoku-oki earthquake[8]. Other color lines and small stars show coseismic slip distributions and epicenters of large megathrust earthquakes[9] ($M \geq 7.0$) that occurred during 1900 to 2011. Panels (**d** to **f**) are the same as panels (**a** to **c**) but for the 2-D residual Vp image of the overriding plate. Other labels are the same as those in **Fig. S1**.

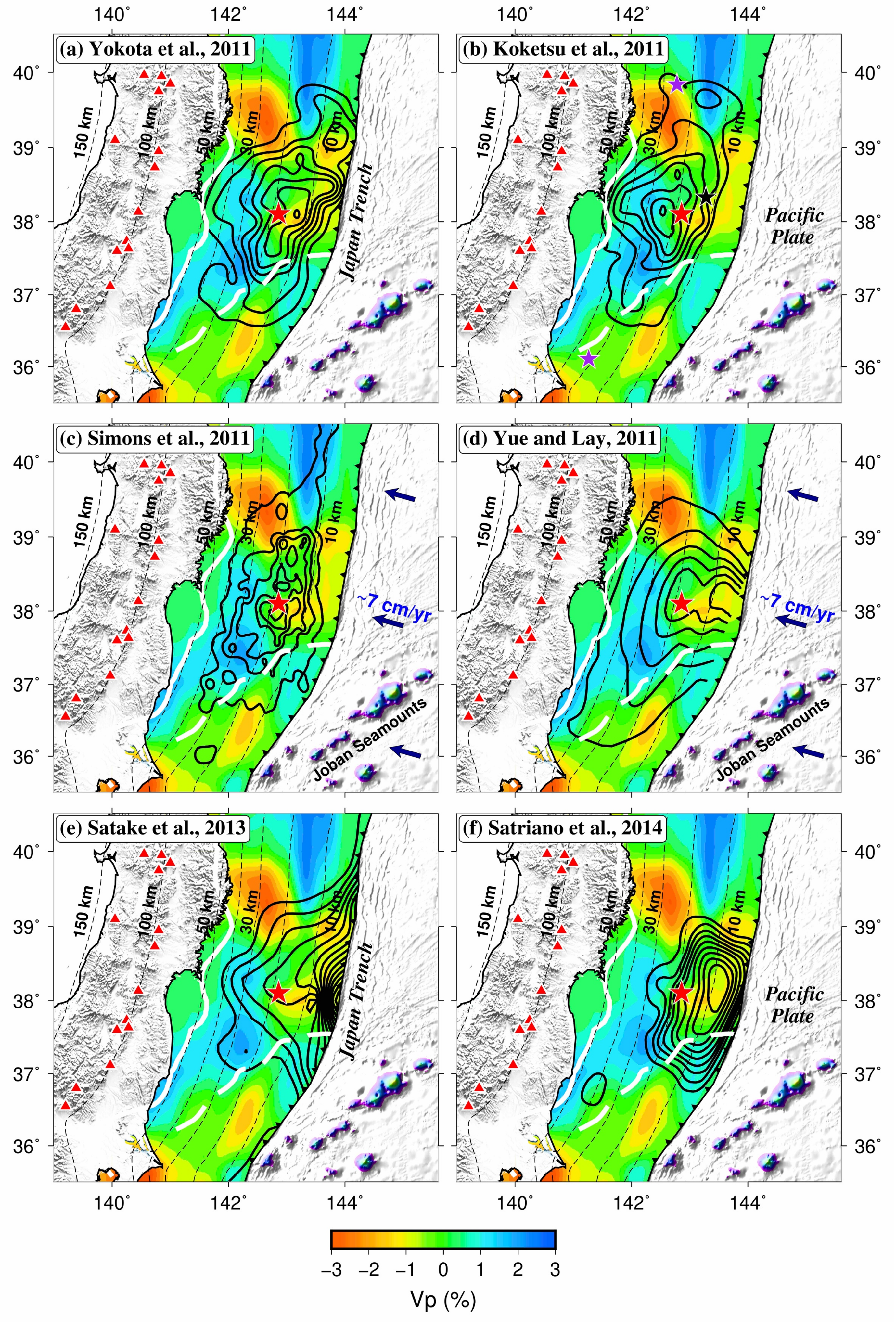
(a) Yokota et al., 2011
(b) Koketsu et al., 2011
(c) Simons et al., 2011
(d) Yue and Lay, 2011
(e) Satake et al., 2013
(f) Satriano et al., 2014
150 km
100 km
50 km
30 km
10 km
Japan Trench
Pacific Plate
~7 cm/yr
Joban Seamounts
140°
142°
144°
36°
37°
38°
39°
40°
−3 −2 −1 0 1 2 3
Vp (%)

Supplementary Figure 12. Coseismic slip models for the 2011 Tohoku-oki earthquake (Mw 9.0). These slip models were determined using different data types and inversion strategies. (a) Yokota et al.[10]; (b) Koketsu et al.[11]; (c) Simons et al.[12]; (d) Yue and Lay[13]; (e) Satake et al.[14]; (f) Satriano et al.[15]. Red and blue colors in each panel denote low and high perturbations, respectively, of residual P-wave velocity (Vp) along the upper boundary of the subducting slab (UBP) determined by this study. The Vp perturbation scale is shown at the bottom. Other labels are the same as those in **Fig. S1**.

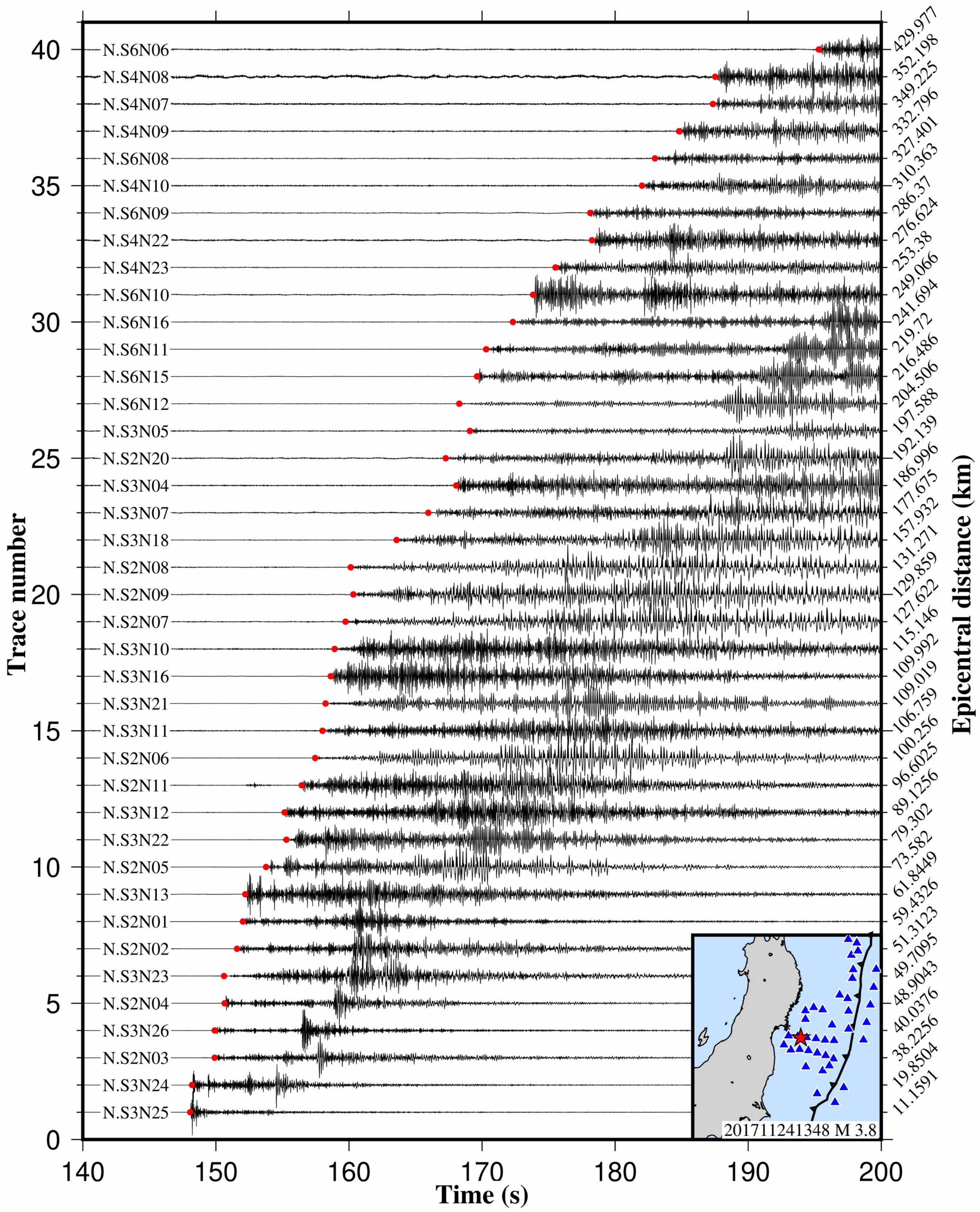

Supplementary Figure 13. Examples of picking P-wave arrival times of a local earthquake recorded at the S-net stations. The waveforms are filtered using a Butterworth filter with a frequency band of 1-10 Hz. The red dots denote the picked P-wave arrival times. The epicenter (red star) and seismic stations (blue triangles) are shown in the inset map.

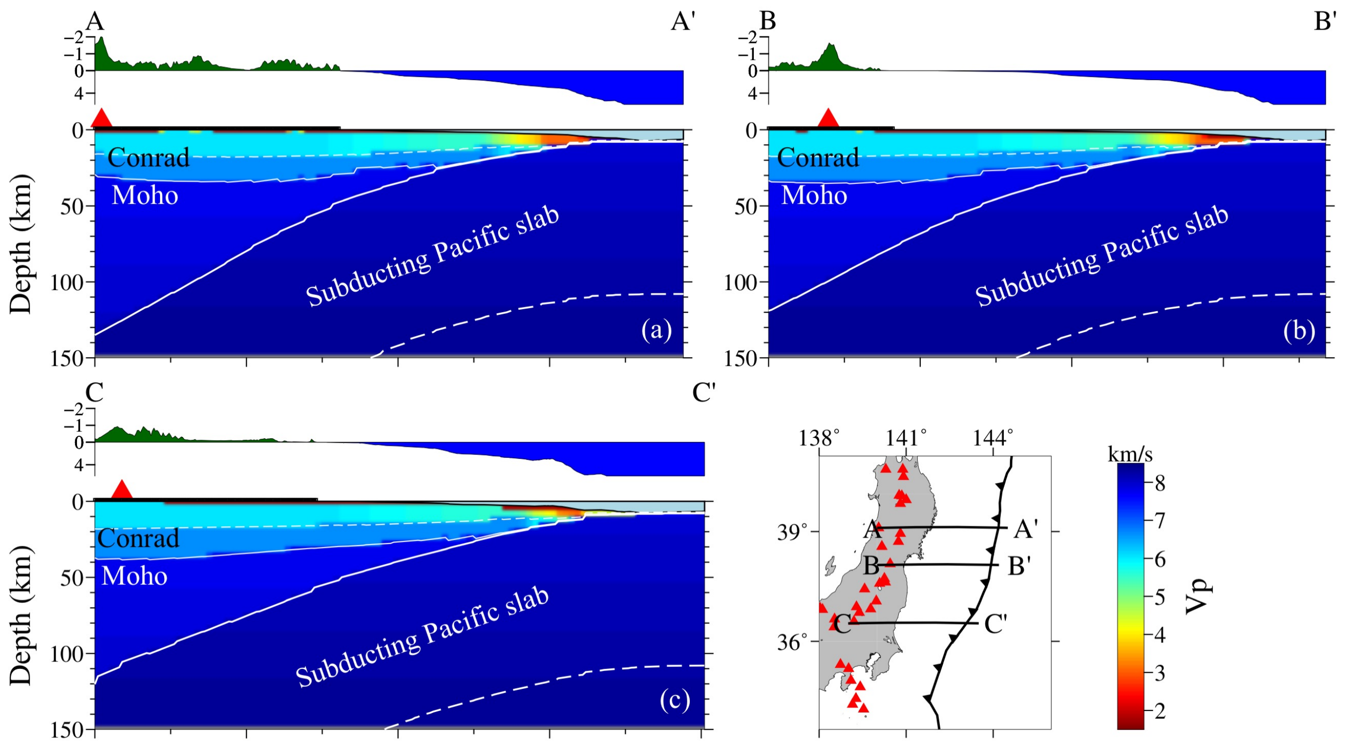

Supplementary Figure 14. East-west vertical cross-sections showing the starting Vp model that is constructed by referring to previous tomographic studies and results of active-source seismic surveys in the Tohoku region. Colors in each profile show absolute P-wave velocity (Vp) whose scale is shown beside the map. Locations of the cross-sections are shown in the inset map.

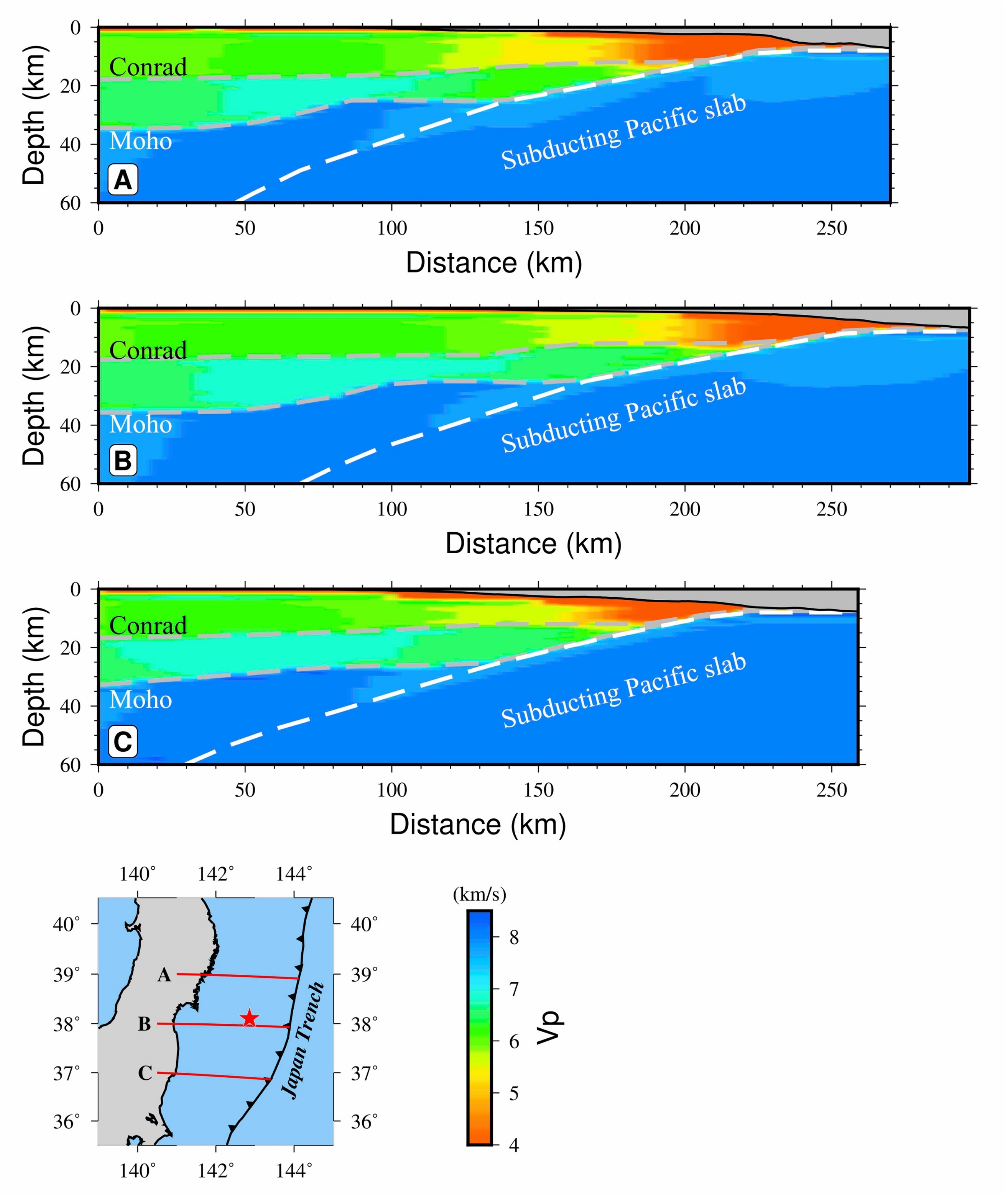

Supplementary Figure 15. Vertical cross-sections of P-wave velocity (Vp) tomography obtained by this study. Red and blue colors denote low and high Vp values, respectively, whose scale is shown beside the map. Locations of the cross-sections are shown in the map. In each panel, the white dashed line denotes the upper boundary of the subducting Pacific slab, whereas the gray dashed lines denote the Conrad and Moho discontinuities.

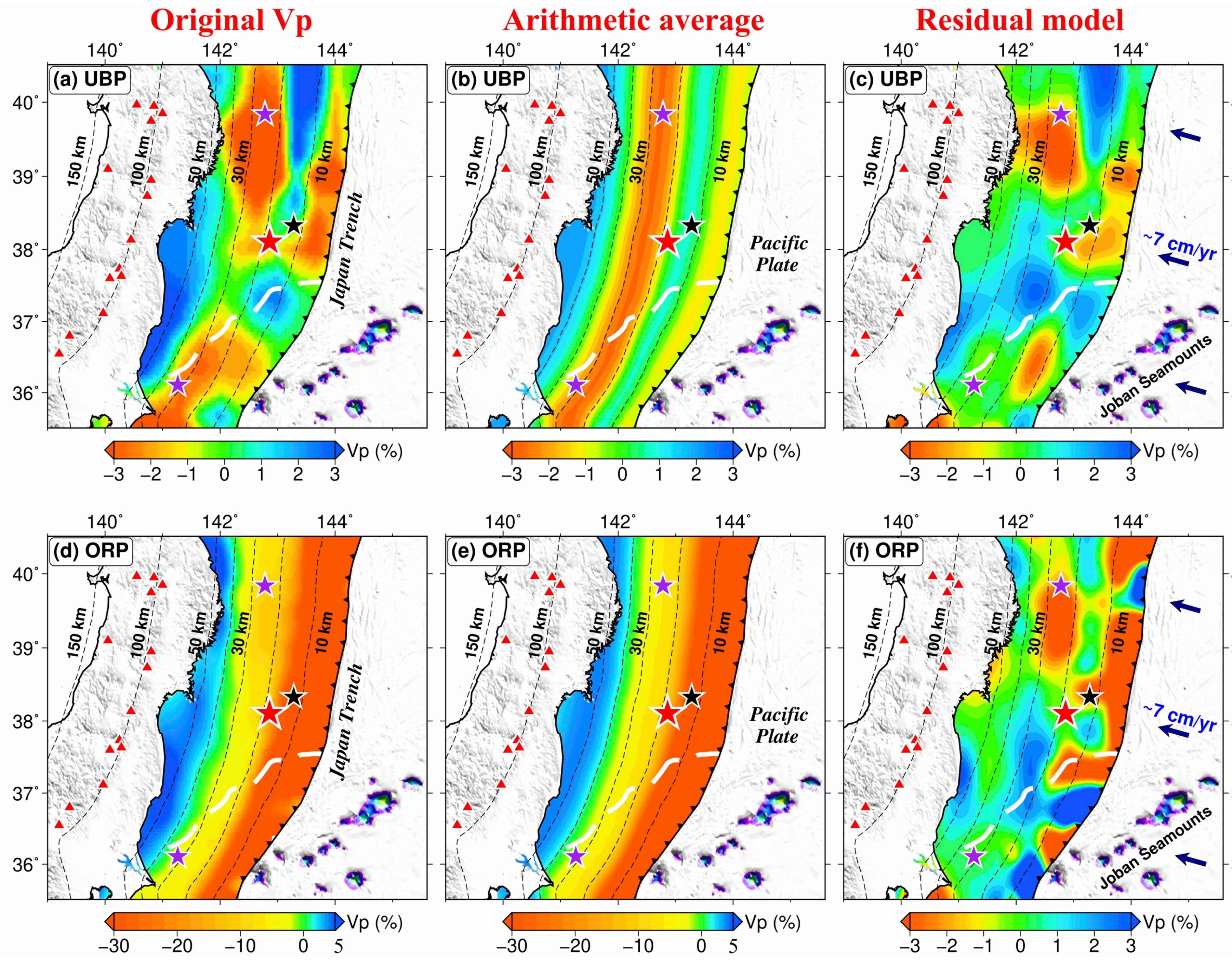

Original Vp
Arithmetic average
Residual model
(a) UBP
(b) UBP
(c) UBP
(d) ORP
(e) ORP
(f) ORP
140°
142°
144°
40°
39°
38°
37°
36°
150 km
100 km
50 km
30 km
10 km
Japan Trench
Pacific Plate
~7 cm/yr
Joban Seamounts
Vp (%)
−3 −2 −1 0 1 2 3
−30 −20 −10 0 5

Supplementary Figure 16. Results of a tomographic inversion with deeper velocity discontinuities. The same as **Fig. S3** but for a tomographic inversion with velocity discontinuities (i.e., the Conrad, Moho and the slab upper boundary) that are 3 km deeper than those used by the present study.

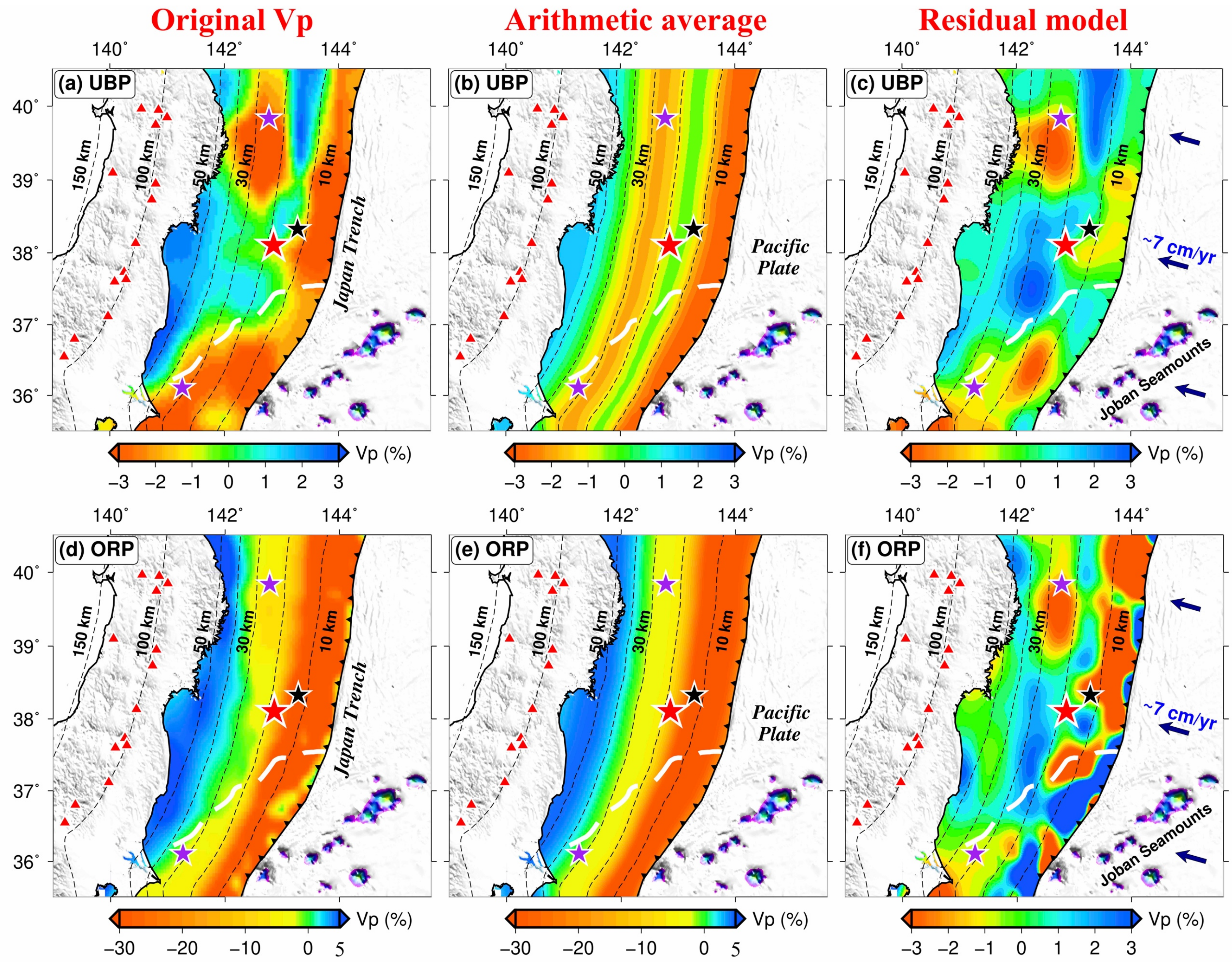

Original Vp
Arithmetic average
Residual model
(a) UBP
(b) UBP
(c) UBP
(d) ORP
(e) ORP
(f) ORP
150 km
100 km
50 km
30 km
10 km
Japan Trench
Pacific Plate
~7 cm/yr
Joban Seamounts
Vp (%)

Supplementary Figure 17. Results of a tomographic inversion using a different starting model. The same as **Fig. S3** but for a tomographic inversion using a different starting velocity model in which Vp is 5.5 km/s for the upper crust and 6.2 km/s for the lower crust (see **Fig. S2**).

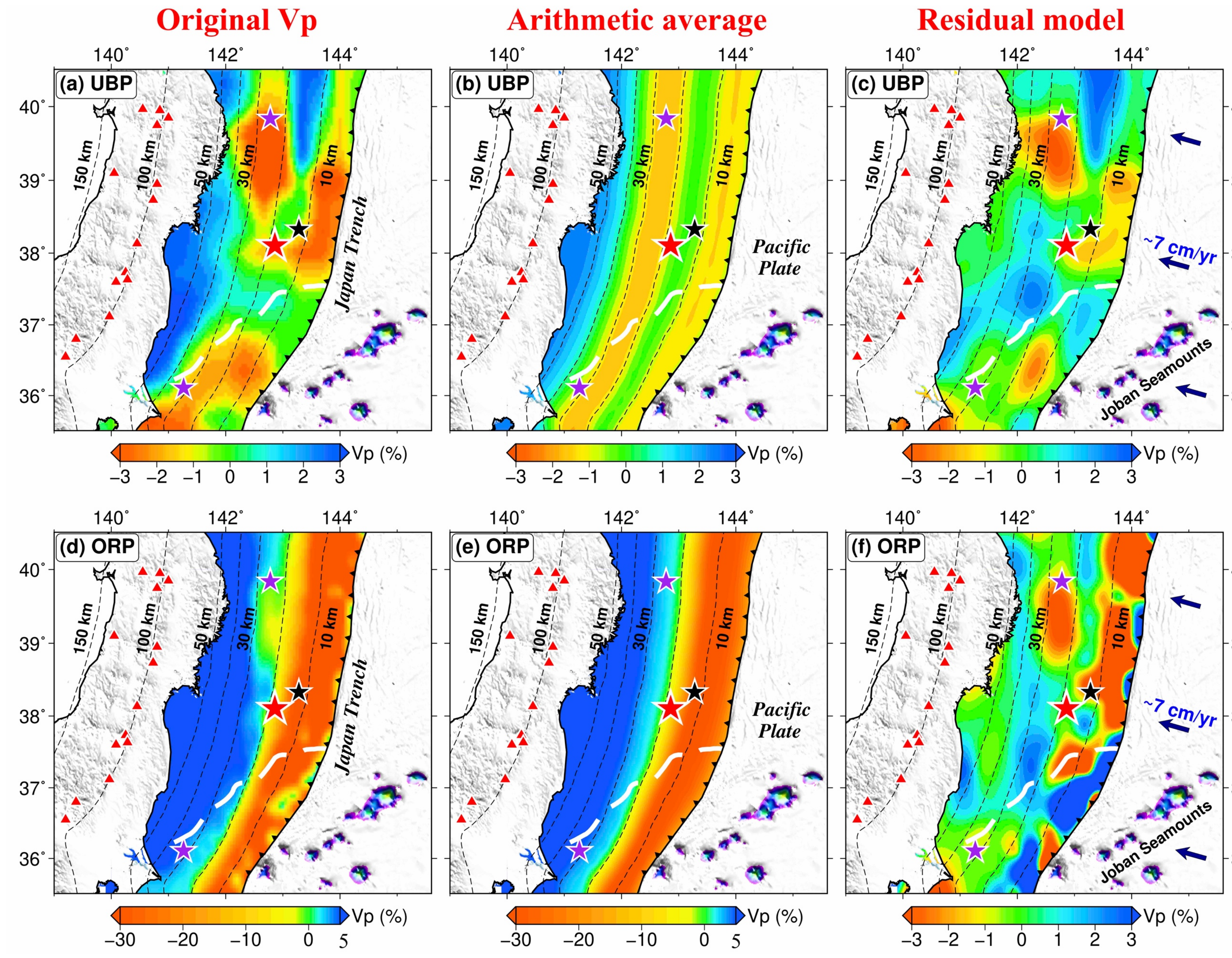

Original Vp
Arithmetic average
Residual model
(a) UBP
(b) UBP
(c) UBP
(d) ORP
(e) ORP
(f) ORP
140°
142°
144°
36°
37°
38°
39°
40°
150 km
100 km
50 km
30 km
10 km
Japan Trench
Pacific Plate
~7 cm/yr
Joban Seamounts
Vp (%)
−3
−2
−1
0
1
2
3
−30
−20
−10
0
5

Supplementary Figure 18. Results of a tomographic inversion with a starting model that includes a low-velocity subducting oceanic crust. The same as **Fig. S3** but for a tomographic inversion with a starting model that includes a low-velocity oceanic crust atop the subducting Pacific slab.

Supplementary References

1. Bassett, D., Sandwell, D. T., Fialko, Y. & Watts, A. B. Upper-plate controls on co-seismic slip in the 2011 magnitude 9.0 Tohoku-oki earthquake. *Nature* **531**, 92–96 (2016).
2. Zhao, D., Huang, Z., Umino, N., Hasegawa, A. & Kanamori, H. Structural heterogeneity in the megathrust zone and mechanism of the 2011 Tohoku-oki earthquake (Mw 9.0). *Geophysical Research Letters* **38**, L17308 (2011).
3. Tomita, F., Kido, M., Ohta, Y., Iinuma, T. & Hino, R. Along-trench variation in seafloor displacements after the 2011 Tohoku earthquake. *Science Advances* **3**, e1700113 (2017).
4. Kurahashi, S. & Irikura, K. Source model for generating strong ground motions during the 2011 off the Pacific coast of Tohoku Earthquake. *Earth, Planets and Space* **63**, 571–576 (2011).
5. Koper, K. D., Hutko, A. R. & Lay, T. Along-dip variation of teleseismic short-period radiation from the 11 March 2011 Tohoku earthquake (Mw 9.0). *Geophysical Research Letters* **38**, L21309 (2011).
6. Ito, Y. *et al.* Episodic slow slip events in the Japan subduction zone before the 2011 Tohoku-Oki earthquake. *Tectonophysics* **600**, 14–26 (2013).
7. Matsuzawa, T., Asano, Y. & Obara, K. Very low frequency earthquakes off the Pacific coast of Tohoku, Japan. *Geophysical Research Letters* **42**, 4318–4325 (2015).
8. Iinuma, T. *et al.* Coseismic slip distribution of the 2011 off the Pacific Coast of Tohoku Earthquake (M 9.0) refined by means of seafloor geodetic data. *Journal of Geophysical Research: Solid Earth* **117**, B07409 (2012).
9. Yamanaka, Y. & Kikuchi, M. Asperity map along the subduction zone in northeastern Japan inferred from regional seismic data. *Journal of Geophysical Research: Solid Earth* **109**, B07307 (2004).
10. Yokota, Y. *et al.* Joint inversion of strong motion, teleseismic, geodetic, and tsunami datasets for the rupture process of the 2011 Tohoku earthquake. *Geophysical Research Letters* **38**, L00G21 (2011).
11. Koketsu, K. *et al.* A unified source model for the 2011 Tohoku earthquake. *Earth and Planetary Science Letters* **310**, 480–487 (2011).
12. Simons, M. *et al.* The 2011 magnitude 9.0 Tohoku-oki earthquake: mosaicking the megathrust from seconds to centuries. *Science* **332**, 1421–1425 (2011).
13. Yue, H. & Lay, T. Inversion of high-rate (1 sps) GPS data for rupture process of the 11 March 2011 Tohoku earthquake (Mw 9.1). *Geophysical Research Letters* **38**, L00G09 (2011).
14. Satake, K., Fujii, Y., Harada, T. & Namegaya, Y. Time and space distribution of coseismic slip

of the 2011 Tohoku earthquake as inferred from tsunami waveform data. *Bulletin of the seismological society of America* **103**, 1473–1492 (2013).

15. Satriano, C. *et al.* Structural and thermal control of seismic activity and megathrust rupture dynamics in subduction zones: Lessons from the Mw 9.0, 2011 Tohoku earthquake. *Earth and Planetary Science Letters* **403**, 287–298 (2014).
